# The plasma exosomal miR-1180-3p serves as a novel potential diagnostic marker for cutaneous melanoma

**DOI:** 10.1186/s12935-021-02164-8

**Published:** 2021-09-20

**Authors:** Yeye Guo, Xu Zhang, Linconghua Wang, Min Li, Minxue Shen, Zhe Zhou, Susi Zhu, Keke Li, Zhiqin Fang, Bei Yan, Shuang Zhao, Juan Su, Xiang Chen, Cong Peng

**Affiliations:** 1 Department of Dermatology, Xiangya Hospital, Central South University, Xiangya Road #87, Changsha, 410008 China; 2 Hunan Key Laboratory of Skin Cancer and Psoriasis, Hunan Engineering Research Center of Skin Health and Disease, Xiangya Hospital, Central South University, Changsha, 410008 China; 3 National Clinical Research Center for Geriatric Disorders, Xiangya Hospital, Central South University, Changsha, 410008 China; 4 Hunan Provincial Key Lab on Bioinformatics, School of Computer Science and Engineering, Central South University, Changsha, 410083 China

**Keywords:** Melanoma, Plasma, Exosome, miR-1180-3p, Diagnostic marker, ST3GAL4

## Abstract

**Background:**

Exosomes are a promising tool in disease detection because they are noninvasive, cost-effective, sensitive and stable in body fluids. MicroRNAs (miRNAs) are the main exosomal component and participate in tumor development. However, the exosomal miRNA profile among Asian melanoma patients remains unclear.

**Methods:**

Exosomal miRNAs from the plasma of melanoma patients (n = 20) and healthy individuals (n = 20) were isolated and subjected to small RNA sequencing. Real-time PCR was performed to identify the differential miRNAs and to determine the diagnostic efficiency. Proliferation, scratch and Transwell assays were performed to detect the biological behavior of melanoma cells.

**Results:**

Exosomal miRNA profiling revealed decreased miR-1180-3p expression as a potential diagnostic marker of melanoma. The validation group of melanoma patients (n = 28) and controls (n = 28) confirmed the diagnostic efficiency of miR-1180-3p. The level of miR-1180-3p in melanoma cells was lower than that in melanocytes. Accordingly, the level of miR-1180-3p was negatively associated with the proliferation, migration and invasion of melanoma cells. Functional analysis and target gene prediction found that ST3GAL4 was a potential target and highly expressed in melanoma tissues and was negatively regulated by miR-1180-3p. Knockdown of ST3GAL4 hindered the malignant phenotype of melanoma cells.

**Conclusions:**

This study indicates that reduced exosomal miR-1180-3p in melanoma patient plasma is a promising diagnostic marker and provides novel insight into melanoma development.

**Supplementary Information:**

The online version contains supplementary material available at 10.1186/s12935-021-02164-8.

## Background

For years, researchers have been devoted to developing liquid biopsy, an ideal approach to molecular cancer diagnosis at an early stage due to its high specificity and noninvasiveness for cancer diagnosis [1]. Circulating tumor cells, DNAs and exosomes are the main targets for liquid biopsy [2]. Among them, exosomes have exceptional advantages in several aspects. Exosomes are 30–150 nm extracellular vesicles secreted by almost all cells and can be detected in various biological fluids [3]. Owing to their tiny size and biocompatibility, exosomes can remain stable in the circulation for a long time [4]. These characteristics make exosomes particularly promising in liquid biopsies [5]. Proteins, metabolites, and nucleic acids are contained in exosomes [6]. MicroRNAs (miRNAs) are noncoding RNAs that are critical exosomal constituents. MiRNAs are processed from precursor molecules (pri-miRNAs) by RNA polymerases II and III, followed by a series of cleavage events [7]. The disorder of miRNAs is associated with various diseases, including cancers, as they regulate the post-transcription of target genes [8] and affect metabolic and cellular pathways, including those related to cell proliferation, differentiation and survival.

Exosomal miRNAs play critical roles in the diagnosis and prognosis of various cancers [9, 10], including melanoma. Cutaneous melanoma is the most severe skin cancer due to its aggressiveness and tendency to metastasize [11]. The five-year survival rate of melanoma varies from over 90% in the localized stage to less than 20% in the advanced stage [12]. When distant metastasis occurs, the median survival is only 6 to 9 months, with a 5-year survival rate of less than 5% [13]. The considerable variation in survival indicates the urgency to screen melanoma patients in the early stages. A miRNA array was generated in plasma-derived exosomes from familial melanoma patients (CDKN2A/p16 gene carriers), nonfamilial melanoma patients and healthy control subjects. Numerous miRNAs have been identified, including miR-17, miR-19a, and miR-21, in metastatic melanoma [14]. The level of exosomal miR-125b in serum was also demonstrated to be relatively high in patients with advanced melanoma. However, most previous studies were performed on Caucasian patients, and exosomal miRNA alterations in Asian melanoma patients remain unknown. The genetic changes, clinical features, anatomical origin and prognosis of melanoma patients vary among different populations [15]. Therefore, identifying differential exosomal miRNAs in the Asian group is highly desirable. In the current study, we described the plasma exosomal miRNA signatures among Chinese melanoma patients to identify a potential diagnostic target and the downstream pathway for this group of patients.

## Methods

### Patient plasma samples

Healthy donors (n = 48) and melanoma patients (n = 48 ) were recruited at Xiangya Hospital, Central South University, following the Declaration of Helsinki. All the donors have signed the consent forms. This study was approved by the Institutional Research Ethics Board of Xiangya Hospital, Central South University. Five milliliters (mL) of whole blood was collected from each subject into ethylenediaminetetraacetic acid (EDTA) tubes and then centrifuged at 3000×*g* for 20 min at 4 ℃. After centrifugation, plasma samples were collected and immediately stored at − 80 °C until use. The clinical characteristics are shown in Additional file 4: Table S1.

### Exosome isolation

Plasma (1.5 mL) was centrifuged at 16,000×*g* for 30 min at 4 °C to remove the microvesicles. The supernatant was subjected to exoRNeasy kits (77144, QIAGEN, Hilden, Germany) according to the manufacturer’s protocol to isolate RNAs from exosomes. Briefly, samples were mixed with Buffer XBP and subjected to an exoEasy spin column. The device was centrifuged for 1 min at 500×*g*, and the column was washed with Buffer XWP. Then, 700 µL of QIAzol was added to the membrane and centrifuged for 5 min at 5000×*g* to collect the lysate. Ninety microliters of chloroform was added to the lysate, shaken, and then incubated for 2 min at room temperature. The sample was centrifuged at 12,000×*g* for 15 min at 4 °C. The upper aqueous phase was collected and mixed with 2 volumes of 100% ethanol and then subjected to an RNeasy MinElute spin column. The sample was centrifuged at ≥ 8000×*g* (≥ 10,000 rpm) for 15 s at room temperature. The spin column was washed with 700 µL of Buffer RWT and 500 µL of Buffer RPEto on an RNeasy MinElute spin column sequentially. Then, 14 µL of RNase-free water was added to the spin column membrane to elute the RNA.

### Cell lines and cell culture

The human melanoma cell lines ME4405, A375, SK-Mel-5 and Sk-Mel-28 (maintained in our laboratory) and the human melanocyte cell line PIG1 (a gift from the Department of Dermatology, Xiangya Third Hospital) were used in this study. Melanoma cells were grown in DMEM (01-052-1A, Biological Industries, Beit HaEmek, Israel) or Opti-MEM for PIG1 (31985070, Gibco, Waltham, MA) supplemented with 10% fetal bovine serum (04-001-1ACS, Biological Industries) and 1% penicillin–streptomycin (03-031-1B, Biological Industries) at 37 °C and 5% CO_2_.

### Cell proliferation assays

Cell viability was assessed using a Cell Counting Kit-8 (CCK-8) assay (Bimake, Houston, TX) according to the manufacturer’s instructions. Cells were seeded into 96-well plates at 3000 cells/well and cultured for 24, 48, 72, or 96 h. Then, 10 μL of CCK-8 solution was added to each well, and the 96-well plate was incubated for 2 h at 37 °C and 5% CO_2_. The fluorescence of each plate was measured using a spectrophotometer at an emission wavelength of 450 nm (Beckman, Brea, CA). Six replicates per sample were analyzed.

### Cell transfection

Cells were transfected with a miR-1180-3p mimic or inhibitor (GeneCopoeia, Rockville, MD) using TurboFect Transfection Reagent (R0533, Thermo Scientific, and incubated for 20 min. The mixture was added to growing cells for 36 to 48 h to facilitate transfection.

### Plasmid and lentiviral vector construction

Lentivirus plasmids containing pLKO.1, pSPAX2 and pMD2G and ST3GAL4 shRNAs were purchased from Thermo Scientific (MA, USA) and GeneChem (Shanghai, China). To establish stable ST3GAL4-knockdown cells, pLKO.1-shST3GAL4 or pLKO.1-shMock plasmids were cotransfected with packaging plasmids (pSPAX2 and PMD2G) into 293T cells. The supernatant fractions containing lentiviral particles were collected separately at 48 and 72 h, and melanoma cells were infected with lentiviral particles in medium supplemented with 10 μg/mL polybrene. At 16 h after infection, the medium was replaced with fresh medium containing a suitable concentration of puromycin. The appropriate experiments were performed with these cells until all control cells (uninfected) were dead (usually 36–48 h) in puromycin-containing medium.

### Transwell assay

For the invasion assay, a Transwell experiment was performed with 8-μm-pore size chambers inserted into 24-well plates (Corning, NY, USA). Matrigel (BD Biosciences, NJ) was diluted (1:7) in serum-free DMEM and then added to each chamber and allowed to solidify completely. Transwell migration assays were performed without Matrigel. Transfected cells were obtained, resuspended in serum-free medium at a concentration of 4 × 10^4^/100 μL and seeded in the upper chambers, while 550 μL of DMEM containing 30% FBS was placed into the bottom chamber as a chemotactic factor. After 24 or 48 h, the cells were fixed with 4% paraformaldehyde for 15 min at room temperature. Nonmotile or noninvaded cells on the top surface of the filter were removed, while motile or invaded cells on the bottom surface were stained with crystal violet. ImageJ software was used to quantify the invaded and migrated cells. The cells in three randomly selected fields (left, middle, right) per well were counted with an inverted microscope system (Ti-S, Nikon, Tokyo, Japan).

### Scratch assay

Cells in complete medium were seeded in a 6-well plate at a density of 1 × 10^5^ cells/well, and a straight line was scratched on the cell monolayer with a 200 μL pipette tip. Then, the cells were washed with PBS three times to remove debris. Finally, the cells were cultured in DMEM supplemented with 10% FBS and imaged at 24 h and 48 h.

### Small RNA sequencing

Small RNA library construction, library purification and sequencing were implemented according to the Wuhan Huada Sequencing Company’s instructions (www.genomics.org.cn, BGI, Shenzhen, China).

### Quantitative real-time PCR analysis

Total RNA was extracted from the cells with TRIzol reagent. Exosomal RNAs were extracted by exoRNeasy kits as described above. One microgram (cells) or 10 ng (exosomes) of total RNA was used as the template for the reverse transcription reaction (miRNA First-Strand cDNA Synthesis Kit for miRNA qPCR array, QP018, GeneCopoeia; SuperScript III First-Strand Synthesis System for Reverse Transcription PCR, 18080051, Invitrogen, Waltham, MA). The PCR primers for miR-1180-3p and U6 were purchased from GeneCopoeia, and the other primers used in this study were as follows:

*ST3GAL4*: Forward *5′GTCGTATTGGAGACCGTCAAG′.*
  Reverse *5′CTTGGAAGGGAGAAAAGGTGAG′.*

*GAPDH*: Forward *5′GTATCGTGGAAGGACTCATGAC.*
  Reverse *5′ACCACCTTCTTGATGTCATCAT.*

Real-time PCR was performed using a miRNA qRT–PCR Detection Kit (QP016, GeneCopoeia) or 2X SYBR Green qPCR Master Mix (B21702, Bimake, Houston, TX). qRT–PCR assays and data collection were performed on a QuantStudio 3 real-time PCR system (Applied Biosystems, Waltham, MA). Data were analyzed using the 2^−△△CT^ values. U6 or GAPDH was used as the control.

### Luciferase reporter gene assays

HEK293T cells were transfected with the pGL3-ST3GAL4-WT or pGL3-ST3GAL4-Del luciferase reporter plasmid (constructed in our lab) and pRLTK, the Renilla luciferase control reporter vector (P100001, Promega, USA), and then treated with the miR-1180-3p mimic or control. After 24 h of transfection, the firefly and Renilla luciferase activities in the cell lysates were analyzed with a dual luciferase assay kit (Promega, Madison, WI) according to the manufacturer’s protocol. For each transfection, the luciferase activities of four replicates were averaged.

### Bioinformatics analysis

The following reads were filtered from the raw data of each sample: (1) low-quality reads, (2) reads with 5′ primer contaminants or without 3′ primers, (3) reads without the insert, (4) reads with poly A bases, and (5) reads shorter than 18 nt. After filtering, the remaining clean data were stored as FASTQ Files. Clean sequencing reads were aligned to the reference genome (hg38) and then annotated by AASRA [16]. The aligned data were mapped to known miRNAs using miRBase version 21. Differential miRNA expression was assessed using DEGseq [17]. Then, random forest was applied to the DEGseq results to identify significantly differentially expressed miRNAs. The target genes of miRNAs were predicted through miRWalk 2.0 [18], ComiR [19], and miR_Pathway [20]. The expression profiles and prognostic significances of target genes were analyzed by GEPIA [21] and the Human Protein Atlas (https://www.proteinatlas.org/).

### Statistical analysis methods

Statistical results are presented as the means ± SDs, and significant differences were analyzed by Student’s t-test or one-way ANOVA. P values < 0.05 were considered statistically significant. Receiver operating characteristic (ROC) curves were constructed using the expression value of miR-1180-3p. The area under the curve (AUC) with the 95% CI was calculated. The Wilcoxon–Mann–Whitney test was used to test the null hypothesis that the AUC was equal to 0.5.

## Results

### Characterization of circulating exosomes in melanoma patients

**Fig. 1:**
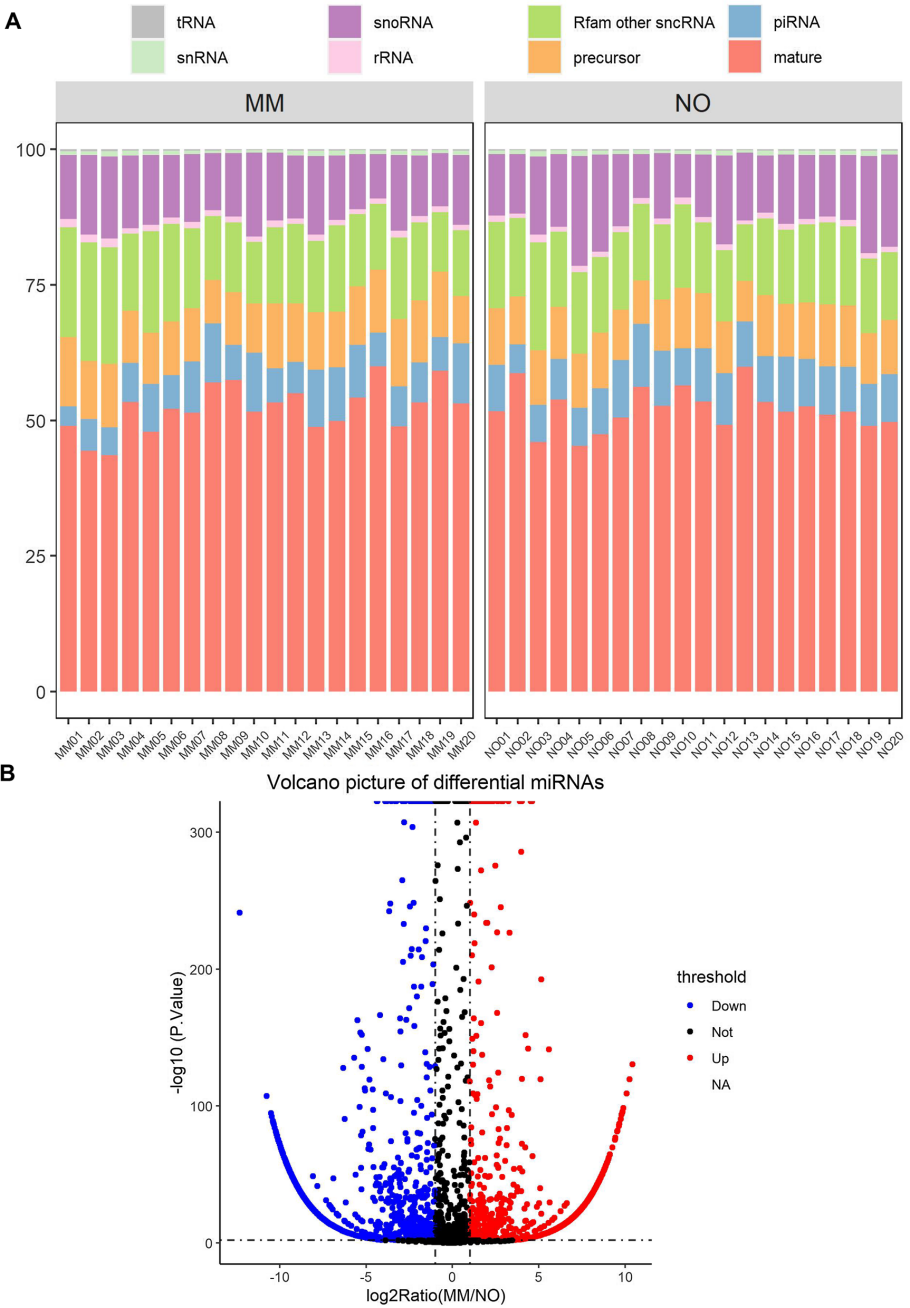
Characterization of circulating exosomes in melanoma. **A** Distribution of mappable small RNAs by small RNA sequencing in exosomes from plasma of melanoma patients (MM, n = 20) and healthy donors (NO, n = 20). Small RNA sequencing was performed as described in Methods. **B** Volcano plot of the differentially expressed exosomal miRNAs between two groups. Significantly downregulated genes are in blue (sig = True), and significantly up-regulated genes are in red (sig = True). Non-significant genes are in black (sig = False). Black vertical lines highlight log fold changes of − 1 and 1, while Black horizontal line represents a padj of 0.001

Exosomal miRNAs from the plasma of melanoma patients (n = 20) and healthy individuals (n = 20) were isolated and then subjected to miRNA sequencing. Over 50% of the mappable RNAs were miRNAs (52.17% in melanoma and 51.99% in healthy donors). The rest of the RNAs were represented as transfer RNAs (tRNAs), small nuclear and nucleolar RNAs (snRNAs and snoRNAs), ribosomal RNAs (rRNAs), Rfam other small noncoding RNAs (sncRNAs), and precursor and Piwi-interacting RNAs (piRNAs), as shown in Fig. 1A. The distributions of exosomal small RNAs in the plasma of melanoma patients and controls were not significantly different. Notably, miRNAs account for a substantial portion of exosomes. Therefore, we focused on the differential miRNAs between melanoma patients and healthy donors that might be predictive of prognosis.

In total, 9703 differential miRNAs, including 666 identified miRNAs and 9037 novel miRNAs, were found by sequencing. The volcano plot describes the 666 identified miRNAs with an adjusted p value < 0.05 (Fig. 1B). The y-axis represents the negative log 10 value (P value), and the x-axis presents the log2 ratio of melanoma patients vs. healthy donors (MM/NO). The upregulated miRNAs are shown as red dots (log2 FC > 1), while the downregulated miRNAs are shown as blue dots (log2 ratio (MM/NO) <  − 1).

**Fig. 2:**
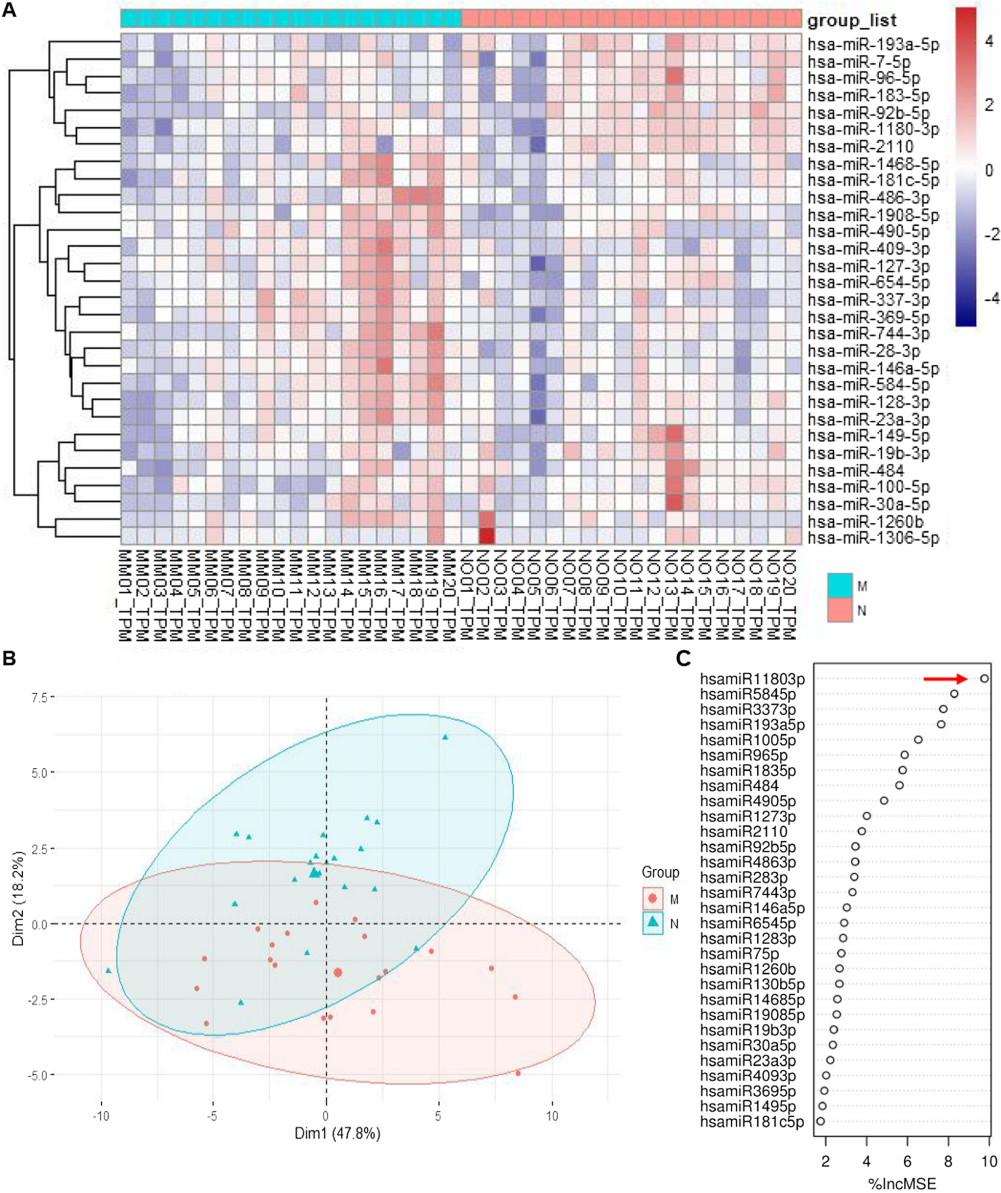
Random Forest analysis of differential miRNAs. **A** Heat map of small RNA sequencing analysis for 30 selected genes by Random Forest algorithm. The heat map shows log10 FPKM values for 30 selected genes (rows) and 40 samples (columns). miRNAs and samples were hierarchically clustered based on Euclidean distance of z-score data and average linkage (dendrogram not shown for samples). **B** Principal component analysis (PCA) of exosomal miRNA expression. PCA for miRNA expression in two groups (30 differentially expressed miRNAs; FDR-adjusted p ≤ 0.05). **C** MiRNAs selection via Random Forest analyses ordered by the importance of contribution towards melanoma development

To better identify the miRNAs, we used random forest (RF) plots to analyze the 666 known miRNAs, revealing 30 significantly altered miRNAs. Using these 30 differential miRNAs, clustering and principal component analysis (PCA) were generated to allocate samples into groups with similar exosomal miRNA expression patterns (Fig. 2A and B). Notably, miR-1180-3p exhibited the highest score in random forest analysis, indicating that it may be vitally important for melanoma development (Fig. 2C).

### miR-1180-3p expression is reduced in exosomes derived from the plasma of melanoma patients and in melanoma cells

**Fig. 3:**
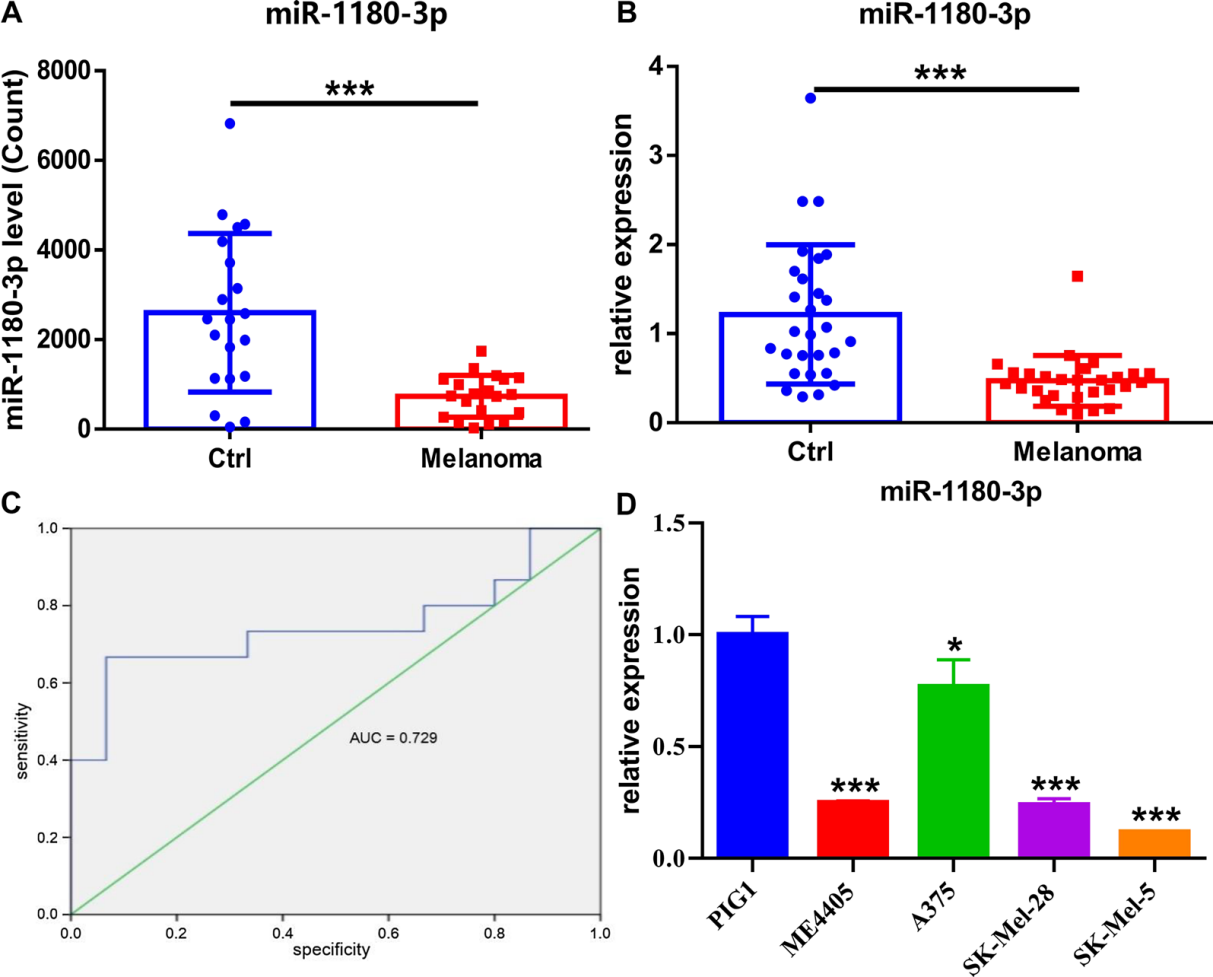
miR-1180-3p is reduced in exosomes from plasma of melanoma patients and in melanoma cells. **A** Exosomal miR-1180-3p is downregulated in melanoma patients. The reads count of miR-1180-3p in small RNA sequencing was detected as described in Methods. Significant difference was evaluated using Student’s t-test, *p < 0.05, **p < 0.01, ***p < 0.001. **B** Exosomal miR-1180-3p is decreased in the validation cohort of melanoma patients. Exosomal RNA was extracted from plasma of melanoma patients (n = 28) and healthy individuals (n = 28), and RT-Q-PCR was performed as described in Methods. Data from multiple experiments are expressed as the mean ± SD (n = 3). Significant differences were evaluated using Student’s t-test, *p < 0.05, ** p < 0.01, ***p < 0.001. **C** ROC curve of miR-1180-3p. The curve was constructed by the expression value for miR-1180-3p. The Area Under the Curve (AUC) with 95% CI was computed and shown for ROC curve. The Wilcoxon-Mann–Whitney test was used to test the null hypothesis that the AUC is equal to .5 (i.e., no predictive power) and the P values for each test were shown. **D** miR-1180-3p is decreased in melanoma cells. Total RNAs were extracted from melanocyte (PIG1) and melanoma cells (ME4405, A375, SK-Mel-28, SK-Mel-5), and RT-Q-PCR was performed as described in Methods. Data from multiple experiments are expressed as the mean ± SD (n = 3). Significant differences were evaluated using one-way ANOVA, *p < 0.05, **p < 0.01, ***p < 0.001

Therefore, we examined the sequencing data of miR-1180-3p, which was decreased by nearly fourfold in melanoma patients compared with the controls (Fig. 3A). To further verify the alteration, we detected the expression of plasma exosomal miR-1180-3p in another validation cohort of participants (n = 56). This result was consistent with the sequencing data, showing that exosomal miR-1180-3p expression was decreased in the plasma of melanoma patients (Fig. 3B). The expression level of exosomal miR-1180-3p in plasma was then detected to examine its diagnostic power for melanoma. Notably, exosomal miR-1180-3p exhibited an area under the ROC curve (AUC) value of 0.729, suggesting its promise as a predictive model (Fig. 3C).

miR-1180-3p is dysregulated in hepatocellular carcinoma [22] and lung adenocarcinoma [23], while its role in melanoma remains unclear. Therefore, the expression level of miR-1180-3p in melanoma cells was assessed by q-PCR. As expected, melanoma cells exhibited lower levels of miR-1180-3p than melanocytes (Fig. 3D).

### Decreased miR-1180-3p expression promotes the malignant phenotype of melanoma cells

**Fig. 4:**
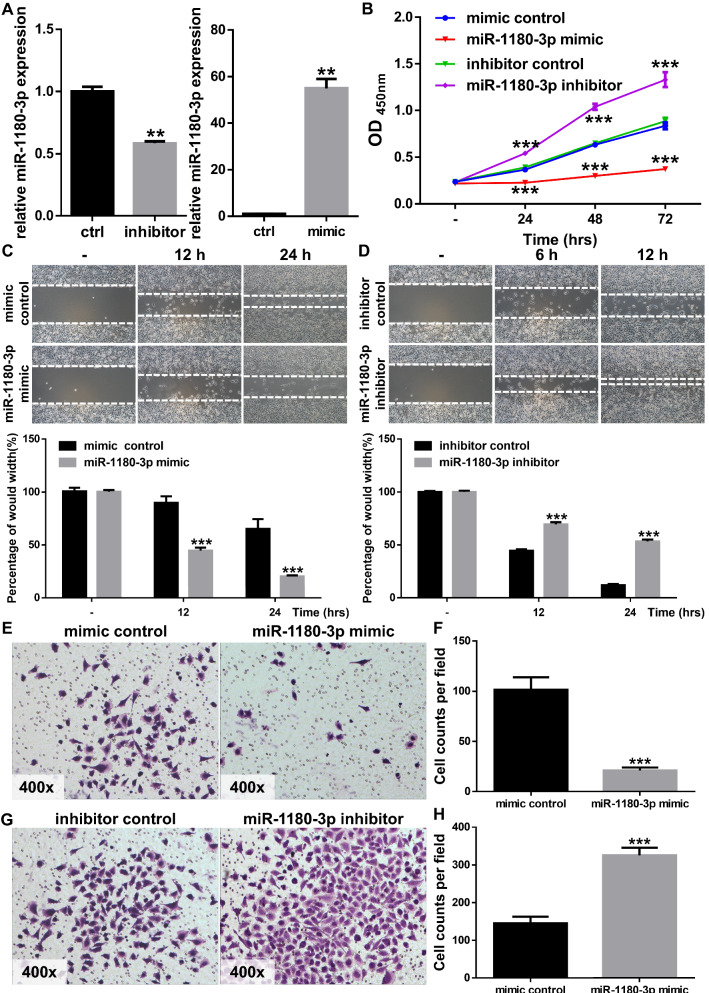
miR-1180-3p negatively regulates melanoma cells’ growth, migration and invasion. **A** Interfere with the expression of miR-1180-3p in melanoma cells. RNAs were extracted from SK-Mel-28 infected with mimic and inhibitor of miR-1180-3p, and RT-Q-PCR was performed as described in Methods. Data from multiple experiments are expressed as the mean ± SD (n = 3). Significant differences were evaluated using Student’s t-test, *p < 0.05, **p < 0.01, ***p < 0.001. **B** miR-1180-3p negatively regulates the growth of melanoma cells. SK-Mel-28 transfected with mimic or inhibitor of miR-1180-3p were seeded into 96-well plates, and cell viability was examined by CCK-8 kit as described in Methods. Data from multiple experiments are expressed as the means ± SD. Significant differences were evaluated using Two-way ANOVA, *p < 0.05, **p < 0.01, ***p < 0.001. **C**, **D** miR-1180-3p regulates melanoma cell migration. The scratch assay was performed as described in Methods. Representative images were taken at indicated hours (**C** and **D** upper panel) and bar chart graphs shown are from three independent experiments (**C** and **D** lower panel). Data are presented as the mean ± SD (n = 3). Significant differences were evaluated using one-way ANOVA, *p < 0.05, **p < 0.01, ***p < 0.001. **E–H** miR-1180-3p regulates the invasion ability of melanoma cells. Transwell assays were performed as described in Methods. Representative images were taken at indicated hours (**E** and **G**). The number of invasive cells per field was calculated, and the data was presented as the means ± SD (n = 4) of each group (**F** and **H**). The significant difference between cells was evaluated by Student’s t-test. *p < 0.05, **p < 0.01, ***p < 0.001

To better understand the biological role of miR-1180-3p, we generated miR-1180-3p-overexpressing or miR-1180-3p-inhibited melanoma cells using an miRNA mimic or inhibitor (Fig. 4A). Interestingly, the growth of melanoma cells was remarkably negatively associated with the level of miR-1180-3p (Fig. 4B), which indicates that miR-1180-3p benefits the growth of melanoma cells. Consistent with this finding, the migration ability of melanoma cells was attenuated by the miR-1180-3p mimics (Fig. 4C). Accordingly, the miR-1180-3p inhibitors enhanced the migration ability of the cells (Fig. 4D). The number of invaded cells was negatively related to the miR-1180-3p expression in melanoma (Fig. 4E–H). These findings support that decreased miR-1180-3p expression promotes the malignant phenotype of melanoma cells.

### MAN2B1 and ST3GAL4 are potential target genes of miR-1180-3p and are negatively regulated by miR-1180-3p

**Fig. 5:**
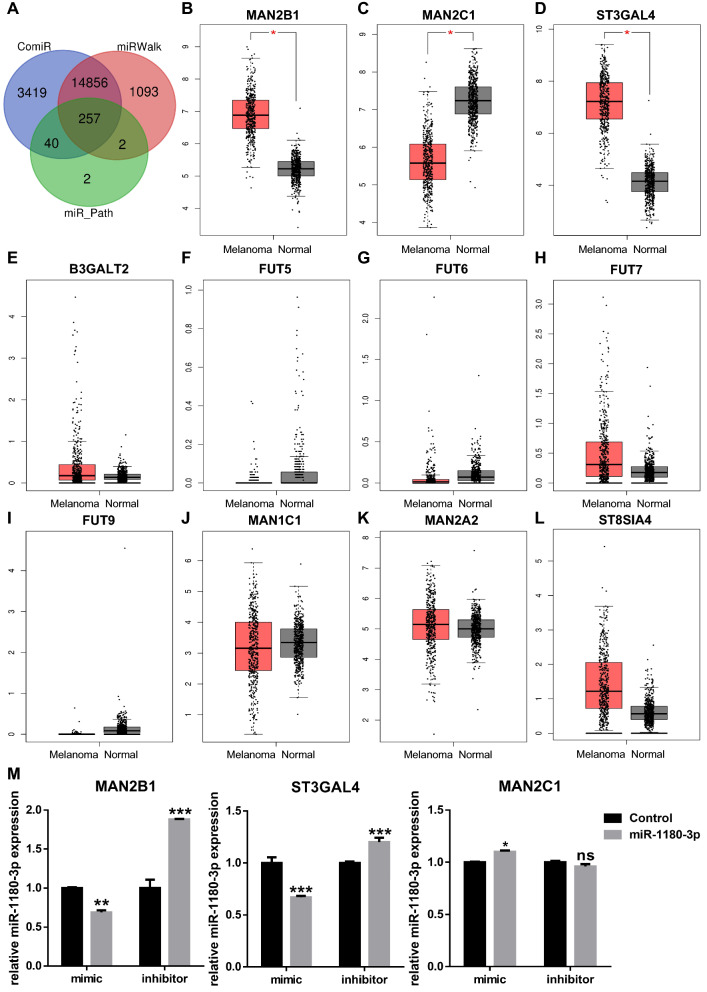
MAN2B1 and ST3GAL4 might be target genes of miR-1180-3p and negatively regulated by miR-1180-3p. **A** Venn diagram of predicted target genes. Target genes predicted by miRWalk, ComiR and miRNA-Pathway were analyzed as described in Methods. **B-L** Validation of MAN2B1, MAN2C1, ST3GAL4 B3GALT2, FUT5, FUT6, FUT7, FUT9, MAN1C1, MAN2A2 and ST8SIA4 from the expression analysis in GEPIA. The box plots are based on 461 melanoma samples (red) and 558 normal samples (gray), revealing the overexpression of MAN2B1 (**B**) and ST3GAL4 (**D**) and the decreased expression of MAN2C1 (**C**) in melanoma. **M** miR-1180-3p negatively regulated the expression of MAN2B1 and ST3GAL4. RNA was extracted from melanoma cells transfected with mimic and inhibitor of miR-1180-3p. RT-Q-PCR was then performed with primers of MAN2B1 (left panel), MAN2C1 (middle panel) and ST3GAL4 (right panel) as described in the Methods. The data from multiple experiments (n = 3) are expressed as the mean ± SD. Significant differences were evaluated using one-way ANOVA, and an asterisk (*) indicates a significant difference (*p < 0.05, **p < 0.01, ***p < 0.001)

It is well established that miRNAs exert their function mainly by base pairing with target mRNAs to negatively regulate their expression [7]. For this reason, we used miRWalk [18], miRNA-Pathway [20] and ComiR [19] to predict the target genes of miR-1180-3p and eventually found 257 intersecting genes (Fig. 5A). Importantly, miR pathway analysis implied that miR-1180-3p is associated with the α-MAN pathway (steps in the glycosylation of mammalian N-linked oligosaccharides) in cutaneous melanoma. Therefore, we focused on 11 genes related to the α-MAN pathway. We further examined the expression profiles of the selected genes by performing gene expression profiling interactive analysis (GEPIA) [21]. Among the 11 genes, MAN2B1, MAN2C1 and ST3GAL4 were significantly altered in melanoma patients compared to the normal controls (Fig. 5B–5D), while the other 8 genes were not significantly different between the patients and healthy controls (Fig. 5E–L). MAN2B1 and ST3GAL4 exhibited higher expression in melanoma patients (Fig. 5B, D), and MAN2C1 was downregulated in tumor tissues (Fig. 5C).

MAN2B1, a member of the alpha-mannosidase family, was reported to be closely related to alpha-mannosidosis [24]. The subcellular localization of mutant MAN2B1 is well known to be associated with inherited metabolic diseases [25], while the role of MAN2B1 in cancers is unknown. In addition to skin cutaneous melanoma (SKCM), the expression of MAN2B1 is generally high in other cancers, including cervical squamous cell carcinoma (CESC), esophageal carcinoma (ESCA), glioblastomas (GBM), kidney renal clear cell carcinoma (KIRC), brain lower-grade glioma (LGG), liver hepatocellular carcinoma (LIHC), and pancreatic adenocarcinoma (PAAD), as shown in Additional file 1: Figure S1A. Notably, MAN2B1 was expressed at the highest level in subjects with melanoma (Additional file 1: Figure S1B). Another alpha-mannosidase, MAN2C1, is downregulated in most tumors except thymoma (THYM), as shown in Additional file 2: Figure S2A. Interestingly, MAN1C1 is known as a tumor suppressor in KIRC [26] and exerts protumor effects on several cancers. In ESCA, inhibition of MAN2C1 suppressed the proliferation of ESCA cells through mitotic arrest and apoptosis [27]. Moreover, MAN2C1 negatively regulates PTEN in prostate cancer, thereby promoting tumor development [28]. Among different cancers, MAN2C1 is moderately expressed in melanoma (Additional file 2: Figure S2B). Alteration of ST3GAL4 expression, a sialyltransferase that transfers sialic acid, has been implicated in carcinogenesis. Interestingly, its expression was dysregulated among different cancers, as shown in Additional file 3: Figure S3A. Interestingly, ST3GAL4 was specifically highly expressed in melanoma (Additional file 3: Figure S3B).

To determine the regulatory impacts of miR-1180-3p on MAN2B1, MAN2C1 and ST3GAL4, we detected the levels of MAN2B1, MAN2C1 and ST3GAL4 in SK-Mel-28 cells with miR-1180-3p overexpression or knockdown. Both MAN2B1 and ST3GAL4 were negatively regulated by miR-1180-3p; however, the expression of MAN2C1 was only slightly increased after the overexpression of miR-1180-3p and was not significantly altered by the miR-1180-3p inhibitor (Fig. 5M). These results suggest that MAN2B1 and ST3GAL4 might be the target genes and negatively regulated by miR-1180-3p.

### MiR-1180-3p binds to ST3GAL4 and regulates the malignant phenotype of melanoma cells

**Fig. 6:**
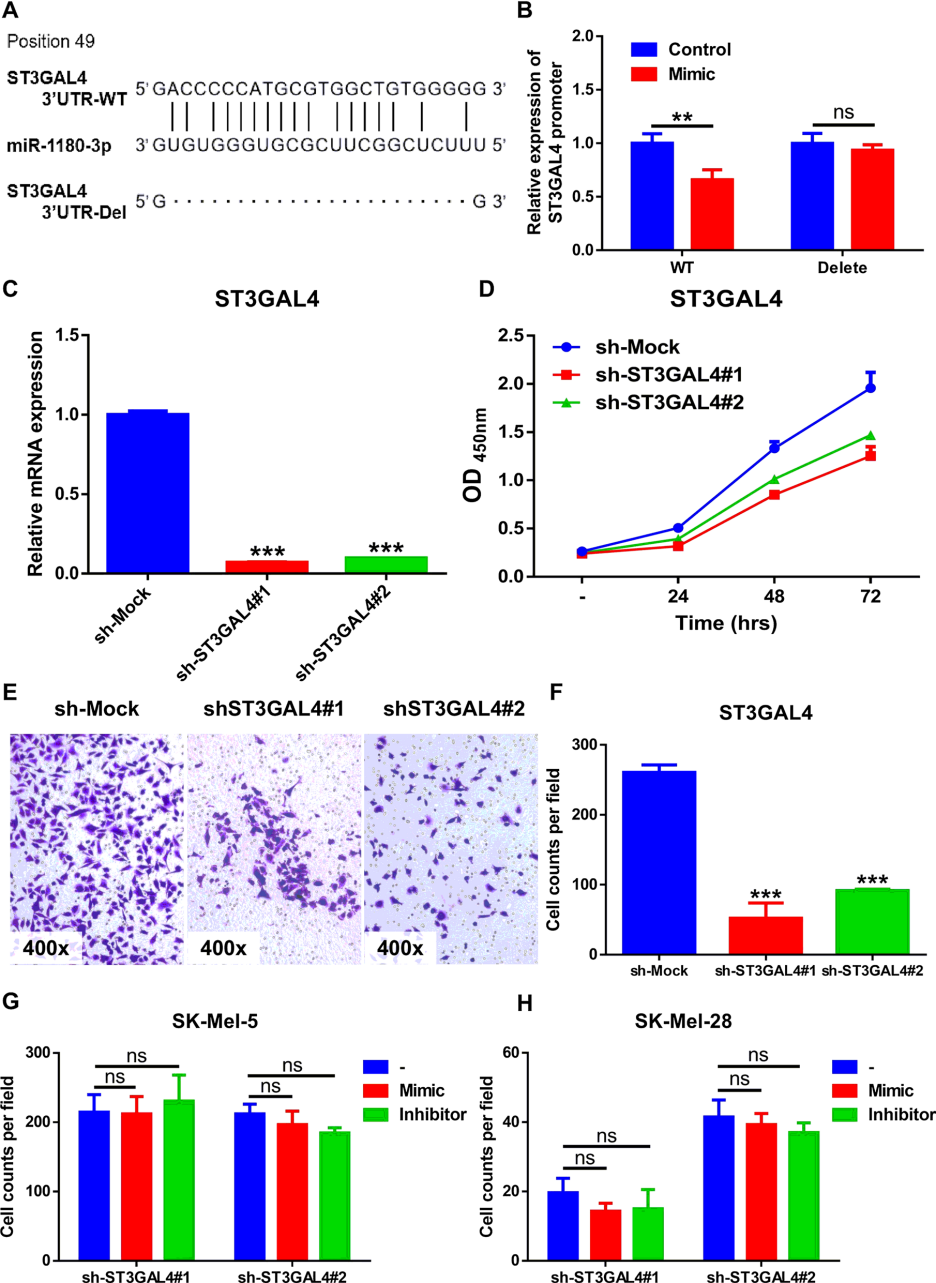
MiR-1180-3p binds to ST3GAL4 and regulate the malignant phenotype of melanoma cells. **A** Schematic diagram of predicted binding sites of ST3GAL4 with miR-1180-3p. The binding sites was predicted by RNAhybrid 2.2 and miRWalk as described in Methods. The predicted binding sites were deleted in the sequence (ST3GAL4-3′UTR-Del) based on ST3GAL4-3′UTR-WT luciferase reporter plasmids. **B** The luciferase activity of ST3GAL4 promoter was inhibited by miR-1180-3p. The luciferase reporter assay was performed in 293T cells transfected with ST3GAL4 3′UTR-WT or ST3GAL4 3′UTR-Del, and treated with miR-1180-3p mimic or control as described in Methods. Data from multiple experiments are expressed as the mean ± SD (n = 4). Significant differences were evaluated using two-way ANOVA, *p < 0.05, **p < 0.01, ***p < 0.001. **C** Knockdown of ST3GAL4 in melanoma cells. Stable knockdown of ST3GAL4 in SK-MEL-28 cells was generated by lentiviral infection and subjected to RT-Q-PCR analysis as described in the Methods. Data from multiple experiments are expressed as the mean ± SD (n = 3). Significant differences were evaluated using Student’s t-test, *p < 0.05, **p < 0.01, ***p < 0.001. **D** Knockdown of ST3GAL4 suppress the proliferation of SK-Mel-28. ST3GAL4-deficient SK-Mel-28 cells were seeded into 96-well plates, and cell viability was examined by CCK-8 kit as described in Methods. Data from multiple experiments are expressed as the means ± SD. Significant differences were evaluated using Two-way ANOVA, *p < 0.05, **p < 0.01, ***p < 0.001. **E**, **F** Inhibition of ST3GAL4 attenuates the invasion ability of SK-Mel-28 cells. Transwell assays were performed as described in Methods. Representative images were taken at indicated hours (**E**). The number of invasive cells per field was calculated, and the data was presented as the means ± SD (n = 4) of each group (**F**). The significant difference between cells was evaluated by Student’s t-test. *p < 0.05, **p < 0.01, ***p < 0.001. **G**, **H** The regulatory role of miR-1180-3p on melanoma cells were diminished after knocking down ST3GAL4. ST3GAL4-deficient melanoma cells were treated with miR-1180-3p mimics or inhibitors. Transwell assays were performed in SK-Mel-5 (**G**) and SK-Mel-28 (**H**) as described in Methods. The number of invasive cells per field was calculated, and the data was presented as the means ± SD (n = 4) of each group. The significant difference between cells was evaluated by Two-way ANOVA. *p < 0.05, **p < 0.01, ***p < 0.001

We then predicted the miR-1180-3p binding sites on MAN2B1 and ST3GAL4 using RNAhybrid 2.2 and miRWalk. The results suggested that miR-1180-3p binds to the 3′UTR′ of ST3GAL4 and to the 5′UTR′ of MAN2B1. Considering that miRNAs are known to typically post-transcriptionally regulate target genes through the 3′-UTR [29], we constructed ST3GAL4 3′UTR-WT and ST3GAL4 3′UTR-Del luciferase reporter plasmids (Fig. 6A). The luciferase reporter assay results suggested that overexpression of miR-1180-3p inhibited the expression of the ST3GAL4 3′UTR-WT promoter but not the ST3GAL4 3′UTR-Del promoter (Fig. 6B), indicating that miR-1180-3p targets ST3GAL4.

To date, few studies have investigated the role of ST3GAL4 in the development of melanoma. Thus, we generated stable ST3GAL4-knockdown melanoma cells using short hairpin RNA (shRNA), as shown in Fig. 6C. The cell proliferation assay displayed inhibited growth after ST3GAL4 knockdown (Fig. 6D). Consistently, the number of migrated cells was remarkably reduced in ST3GAL4-deficient melanoma cells (Additional file 5: Figure S4). In addition, the invasion ability was attenuated when the expression of ST3GAL4 was inhibited in melanoma cells (Fig. 6E, F) (Additional file 5: Figure S4).

As miR-1180-3p might regulate the biological behavior of melanoma cells by targeting ST3GAL4, we treated ST3GAL4-deficient melanoma cells with miR-1180-3p mimics and inhibitors. As expected, the Transwell assay showed no significant difference in the numbers of invaded cells treated with the miR-1180-3p mimics or inhibitors after knocking down ST3GAL4 (Fig. 6G, H) (Additional file 6: Figure S5), supporting our hypothesis that miR-1180-3p regulates the malignant phenotype of melanoma by targeting ST3GAL4.

Taken together, our study results demonstrated that miR-1180-3p expression was decreased in exosomes derived from melanoma patients compared with those from healthy donors and was thus identified as a prospective melanoma biomarker. In addition, decreased miR-1180-3p expression promoted the development of melanoma by targeting ST3GAL4.

## Discussion

In this study, the exosomal miRNA expression profile from the plasma of melanoma patients was investigated. MiR-1180-3p expression was decreased in exosomes derived from melanoma patient plasma and exhibited diagnostic potential. In addition, decreased miR-1180-3p expression promoted the proliferation of melanoma cells by elevating ST3GAL4. To the best of our knowledge, this is the first plasma exosomal miRNA study on Chinese melanoma patients. These findings suggest a prospective strategy for the early diagnosis of melanoma and provide additional insight into melanoma development.

Exosomal miRNAs from plasma have been studied in various cancers with the aim of early diagnosis and improving knowledge of cancer biology. In breast cancer, certain exosomal microRNAs are upregulated in the plasma of patients, including miR-21 and miR-1246 [30]. For pancreatic cancer, exosomal miR-196a and miR-1246 are promising biomarkers [31]. In some cases, plasma exosomal miRNAs serve as markers for differential diagnosis. For instance, miR-21 and miR-181a in plasma exosomes distinguish follicular from papillary thyroid cancer [32]. Different combinations of exosomal miRNAs differentiate adenocarcinoma from squamous cell carcinoma in the early diagnosis of NSCLC [33]. Exosomal miRNAs also have prognostic potential in cancer patients. Among different stages of colorectal cancer, increased miR-21 expression indicates metastasis and a poor prognosis [34]; higher levels of exosomal miR-1290 and miR-375 have been associated with poor overall survival in castration-resistant prostate cancer [35].

Attempts have been made to investigate the functions of exosomal miRNAs in melanoma. Exosomal miR-222 was shown to promote melanoma tumorigenesis [36]. Susan et al. identified several differentially regulated miRNAs derived from the plasma exosomes of patients with sporadic melanoma. However, the study did not show the efficacies of those miRNAs to diagnose melanoma. In addition, the differential miRNAs were analyzed by an miRNA array that included ~ 700 human miRNAs, and some important miRNAs might have been excluded [14]. An miRNA array was assembled that included exosomes derived from melanoma cell lines and indicated that specific miRNAs, including miR-31, miR-186, and miR-34b, are associated with melanoma invasion [37]. Here, our findings revealed the exosomal miRNA profile in the plasma of melanoma patients and revealed reduced miR-1180-3p expression as a potential diagnostic marker for melanoma for the first time.

Studies have revealed the potential of miR-1180-3p in cancer diagnosis. The expression of miR-1180-3p in the blood was found to separate NSCLC patients from healthy individuals, with an AUC of 0.69 [38]. The circulatory plasma expression of miR-1180-3p, miR-425-5p, miR-122-5p, miR-24-3p and miR-4632-5p serves as a biomarker panel for the early detection of gastric cancer [39]. However, the function and mechanism of miR-1180-3p in melanoma have not yet been fully investigated. In the current study, we demonstrated that decreased miR-1180-3p expression supports the growth of melanoma cells and provide new insight into the role of miR-1180-3p in melanoma development.

The incidence rate of melanoma has increased by 110.3% in the last two decades in China [40]. For Asian melanoma patients, acral lentiginous melanoma, the most common subtype, accounts for 50% to 58% of cutaneous melanoma cases [41–43]. Usually, acral melanoma presents in the palms, soles, and nail apparatuses. Therefore, early tumors are difficult to notice and are typically diagnosed at a late stage, resulting in a poor prognosis [44]. Compared to the early stages of melanoma, advanced melanoma causes a significant economic burden [45, 46]. These characteristics suggest that the early and timely diagnosis of melanoma is critical for improving prognosis and will help to reduce the global disease burden. Our findings herein demonstrate that plasma exosomal miR-1180-3p is a promising diagnostic biomarker for melanoma patients. Importantly, the miR-1130-3p/ST3GAL4 axis could be a therapeutic target for melanoma treatment.

## Supplementary Information

**Additional file 1: Figure S1.** MAN2B1 is generally highly expressed in cancers. (**A**) MAN2B1 is generally highly expressed in cancers. The expression profile of MAN2B1 in different cancers was analyzed by GEPIA. MAN2B1 is overexpressed in CESC, DLBC, ESCA, GBM, KIRC, KIRP, LAML, LGG, LIHC, PAAD, SKCM, TGCT, UCEC and UC. (**B**) The expression level of MAN2B1 in melanoma is higher than that in other cancers. Comparison of MAN2B1 expression in different cancers was analyzed in ProteinAtlas as described in Methods.

**Additional file 2: Figure S2.** MAN2C1 is generally down-regulated in cancers. (**A**) MAN2C1 is generally down-regulated in cancers. The expression profile of MAN2C1 in different cancers was analyzed by GEPIA. MAN2B1 is overexpressed in ACC, BRCA, CESC, COAD, ESCA, GBM, LUAD, LUSC, OV, PRAD, READ, SKCM, STAD, TGCT, THCA, UCEC and UCS, but highly expressed in THYM. (**B**) The expression level of MAN2B1 in melanoma is moderate among different cancers. Comparison of MAN2C1 expression in different cancers was analysed in ProteinAtlas as described in Methods.

**Additional file 3: Figure S3.** ST3GAL4 is specifically highly expressed in melanoma. (**A**) ST3GAL4 is specifically highly expressed in melanoma. The expression profile of ST3GAL4 in different cancers was analyzed by GEPIA. ST3GAL4 is overexpressed in SKCM, LAML and PAAD, while down-regulated in DLBC, ESCA, KIRP, OV, TGCT and THYM. (**B**) The expression level of ST3GAL4 in melanoma is significantly higher than that in other cancers. Comparison of ST3GAL4 expression in different cancers was analyzed in ProteinAtlas as described in Methods.

**Additional file 4: Table S1.** The demographic and clinical information of participants.

**Additional file 5: Figure S4.** ST3GAL4 regulates the migration ability of melanoma cells. (**A**) Knockdown of ST3GAL4 inhibit the migration of SK-MEL-28 cells. Migration assays were performed as described in Methods. Representative images were taken at indicated hours. (**B**) Bar chart of migration assay. The number of invasive cells per field was calculated, and the data was presented as the means ± SD (n = 4) of each group. The significant difference between cells was evaluated by Student’s t-test. *p < 0.05, **p < 0.01, ***p < 0.001.

**Additional file 6: Figure S5.** Knockdown of ST3GAL4 impairs the regulatory role of miR-1180-3p on invasion ability of melanoma cells. (**A**-**B**) ST3GAL4-deficient melanoma cells were treated with miR-1180-3p mimics or inhibitors. Representative images of SK-Mel-5 (**A**) and SK-Mel-28 (**B**) were taken at indicated hours as described in Methods.

## Data Availability

The datasets generated and/or analysed during the current study are available from the corresponding author on request.

## Abbreviations

CESC: Cervical squamous cell carcinoma
ESCA: Esophageal carcinoma
GBM: Glioblastomas
KIRC: Kidney renal clear cell carcinoma
LGG: Brain lower-grade glioma
LIHC: Liver hepatocellular carcinoma
miRNA: MicroRNA
PAAD: Pancreatic adenocarcinoma
