# Supplementary figures and images for: The plasma exosomal miR-1180-3p serves as a novel potential diagnostic marker for cutaneous melanoma
Source: Cancer Cell Int. 2021 Sep 20;21:487. doi: 10.1186/s12935-021-02164-8 (PMC8454000; doi:10.1186/s12935-021-02164-8)

# Supplementary Figure 1

A

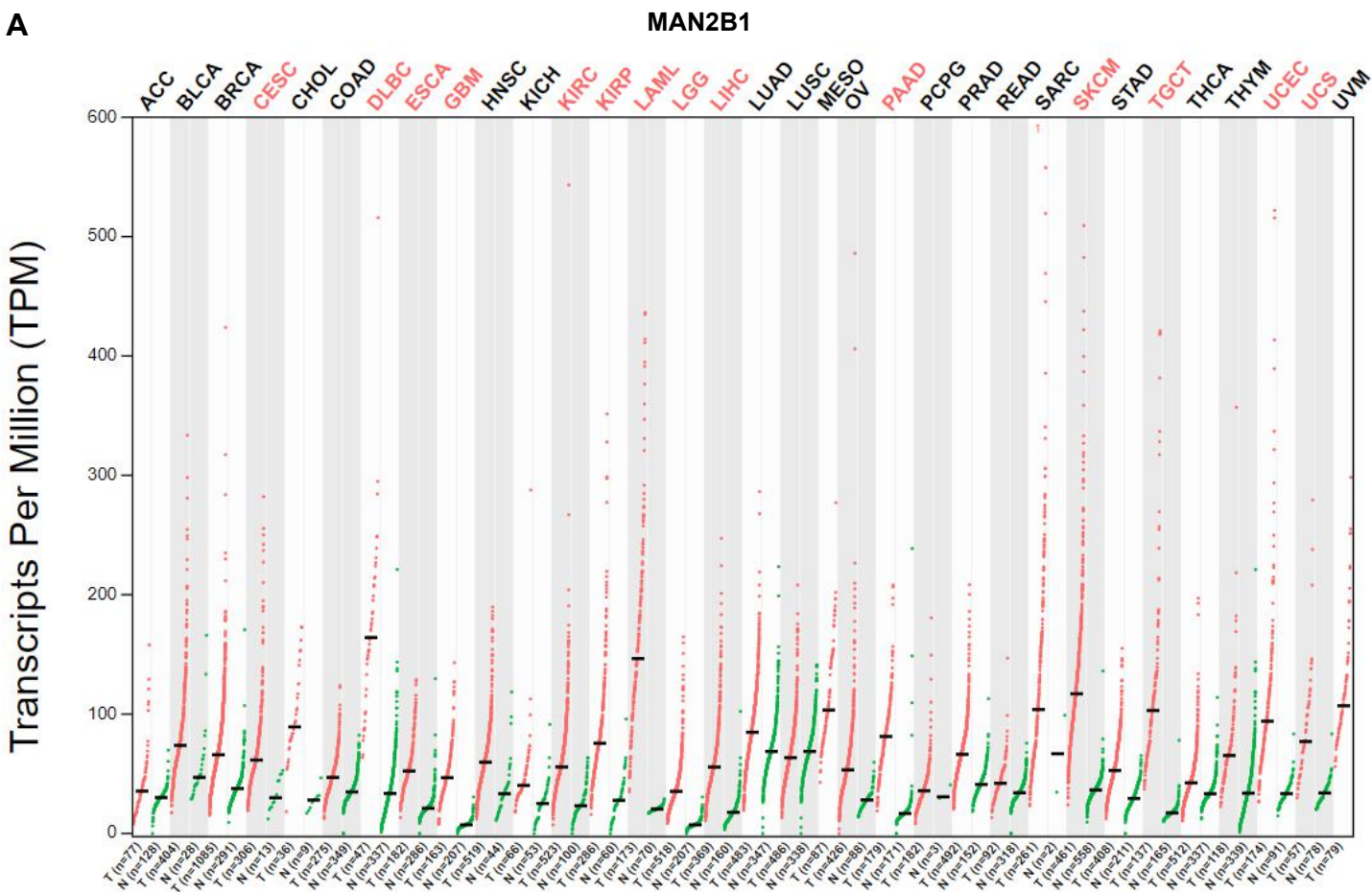

B

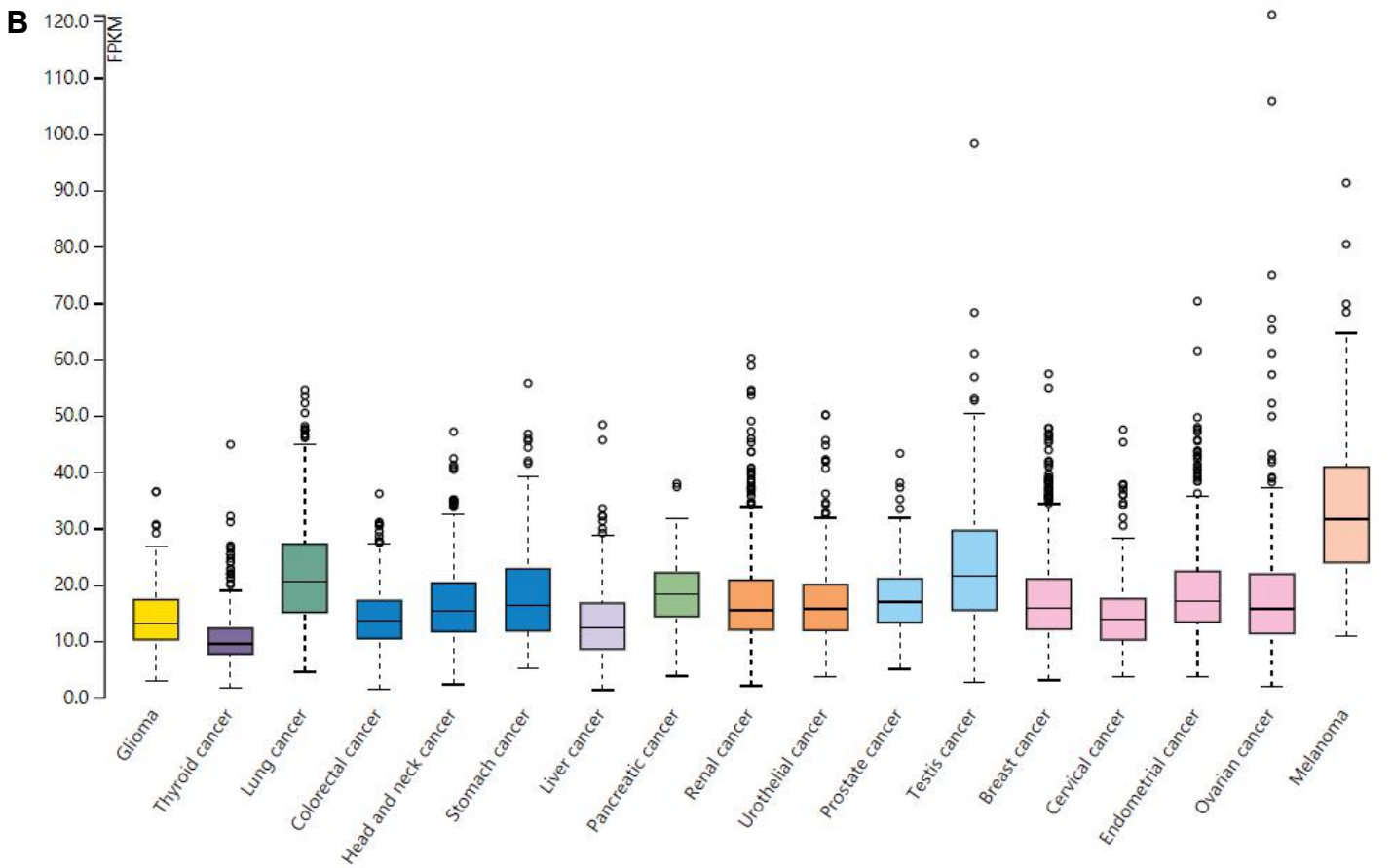

Supplement: Supplementary file 1 — Additional file 1: Figure S1. MAN2B1 is generally highly expressed in cancers. (A) MAN2B1 is generally highly expressed in cancers. The expression profile of MAN2B1 in different cancers was analyzed by GEPIA. MAN2B1 is overexpressed in CESC, DLBC, ESCA, GBM, KIRC, KIRP, LAML, LGG, LIHC, PAAD, SKCM, TGCT, UCEC and UC. (B) The expression level of MAN2B1 in melanoma is higher than that in other cancers. Comparison of MAN2B1 expression in different cancers was analyzed in ProteinAtlas as described in Methods. [file 12935_2021_2164_MOESM1_ESM.pdf]

# Supplementary Figure 2

A

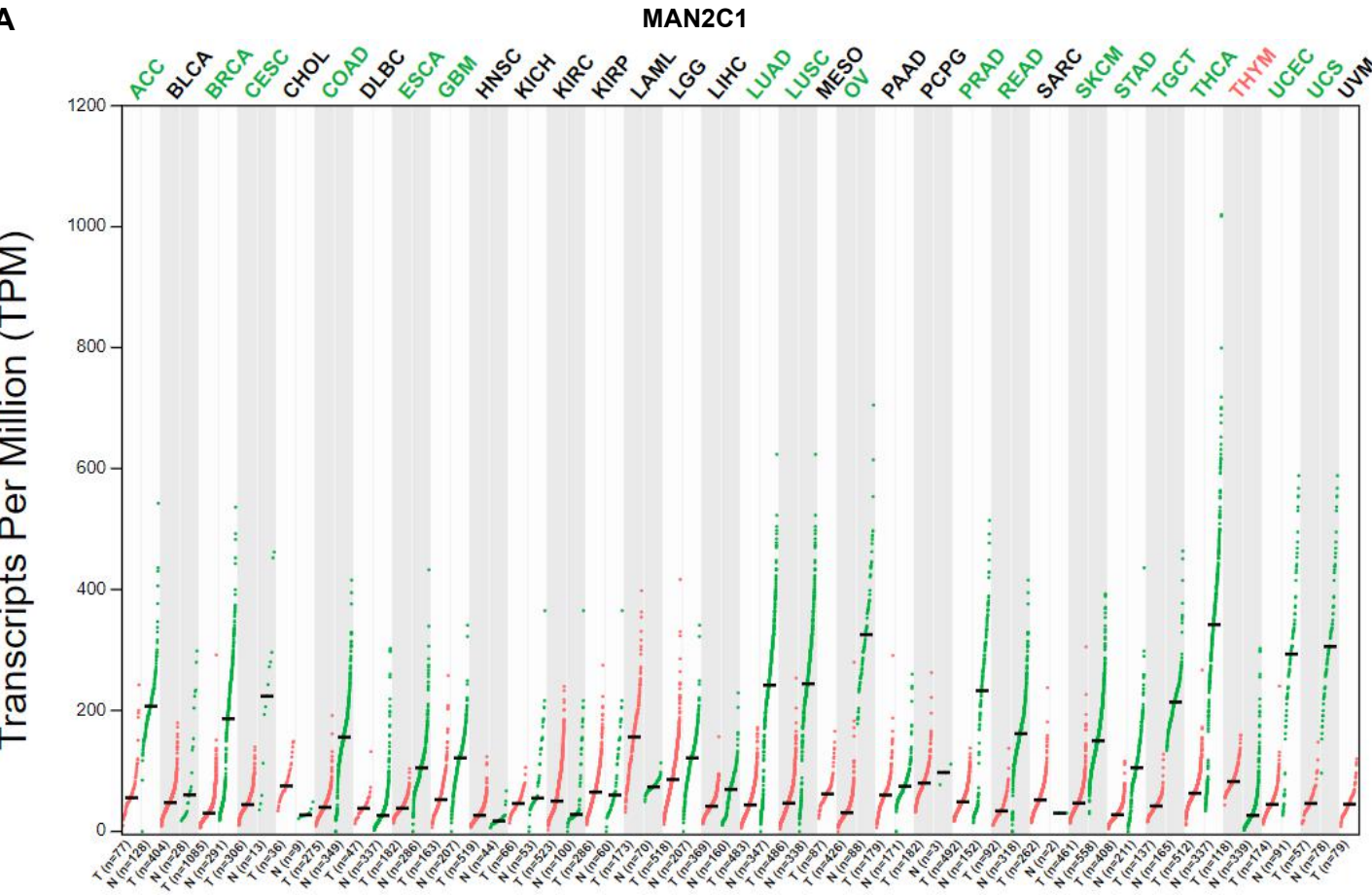

B

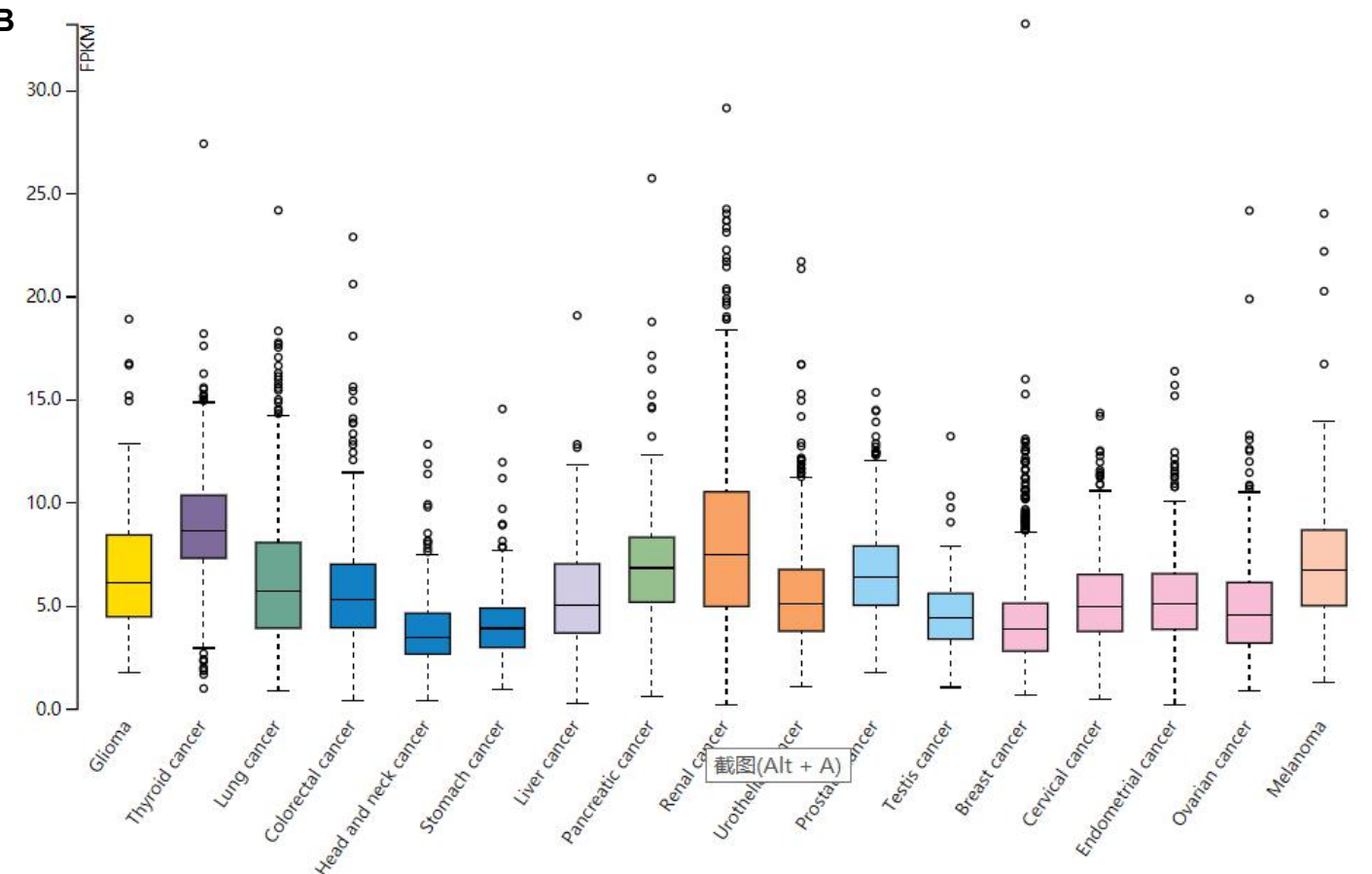

Supplement: Supplementary file 2 — Additional file 2: Figure S2. MAN2C1 is generally down-regulated in cancers. (A) MAN2C1 is generally down-regulated in cancers. The expression profile of MAN2C1 in different cancers was analyzed by GEPIA. MAN2B1 is overexpressed in ACC, BRCA, CESC, COAD, ESCA, GBM, LUAD, LUSC, OV, PRAD, READ, SKCM, STAD, TGCT, THCA, UCEC and UCS, but highly expressed in THYM. (B) The expression level of MAN2B1 in melanoma is moderate among different cancers. Comparison of MAN2C1 expression in different cancers was analysed in ProteinAtlas as described in Methods. [file 12935_2021_2164_MOESM2_ESM.pdf]

# Supplementary Figure 3

A

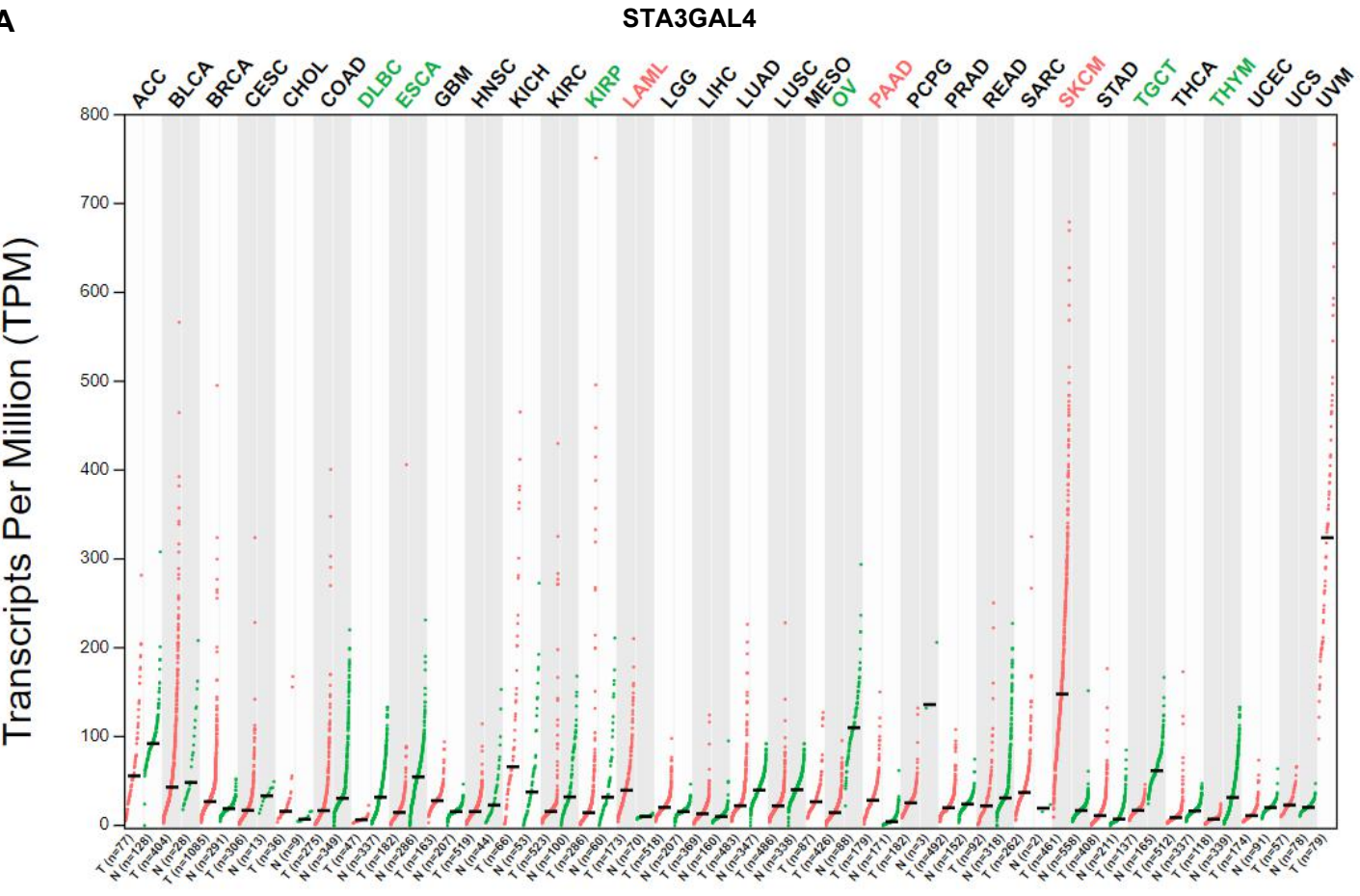

B

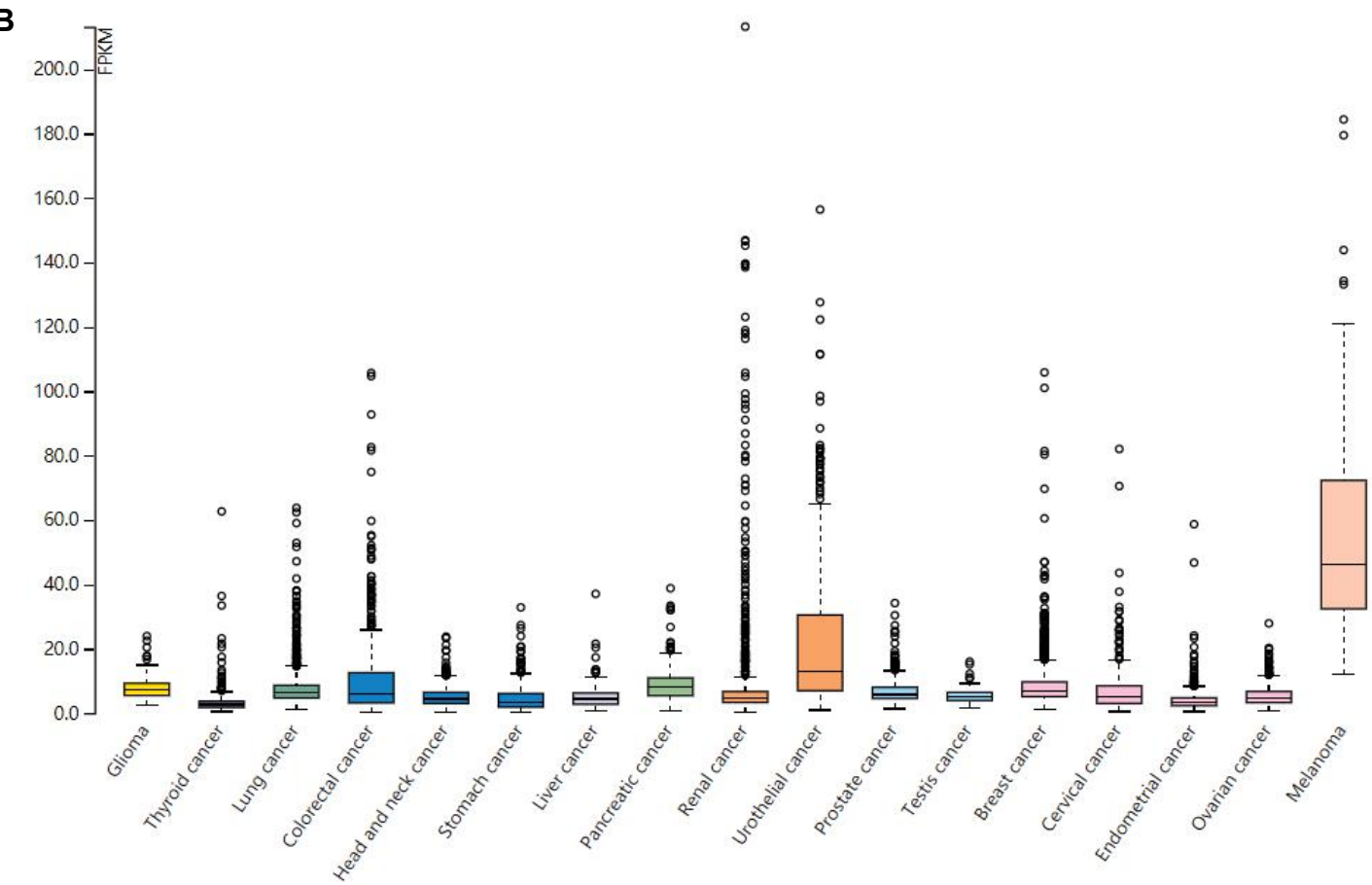

Supplement: Supplementary file 3 — Additional file 3: Figure S3. ST3GAL4 is specifically highly expressed in melanoma. (A) ST3GAL4 is specifically highly expressed in melanoma. The expression profile of ST3GAL4 in different cancers was analyzed by GEPIA. ST3GAL4 is overexpressed in SKCM, LAML and PAAD, while down-regulated in DLBC, ESCA, KIRP, OV, TGCT and THYM. (B) The expression level of ST3GAL4 in melanoma is significantly higher than that in other cancers. Comparison of ST3GAL4 expression in different cancers was analyzed in ProteinAtlas as described in Methods. [file 12935_2021_2164_MOESM3_ESM.pdf]

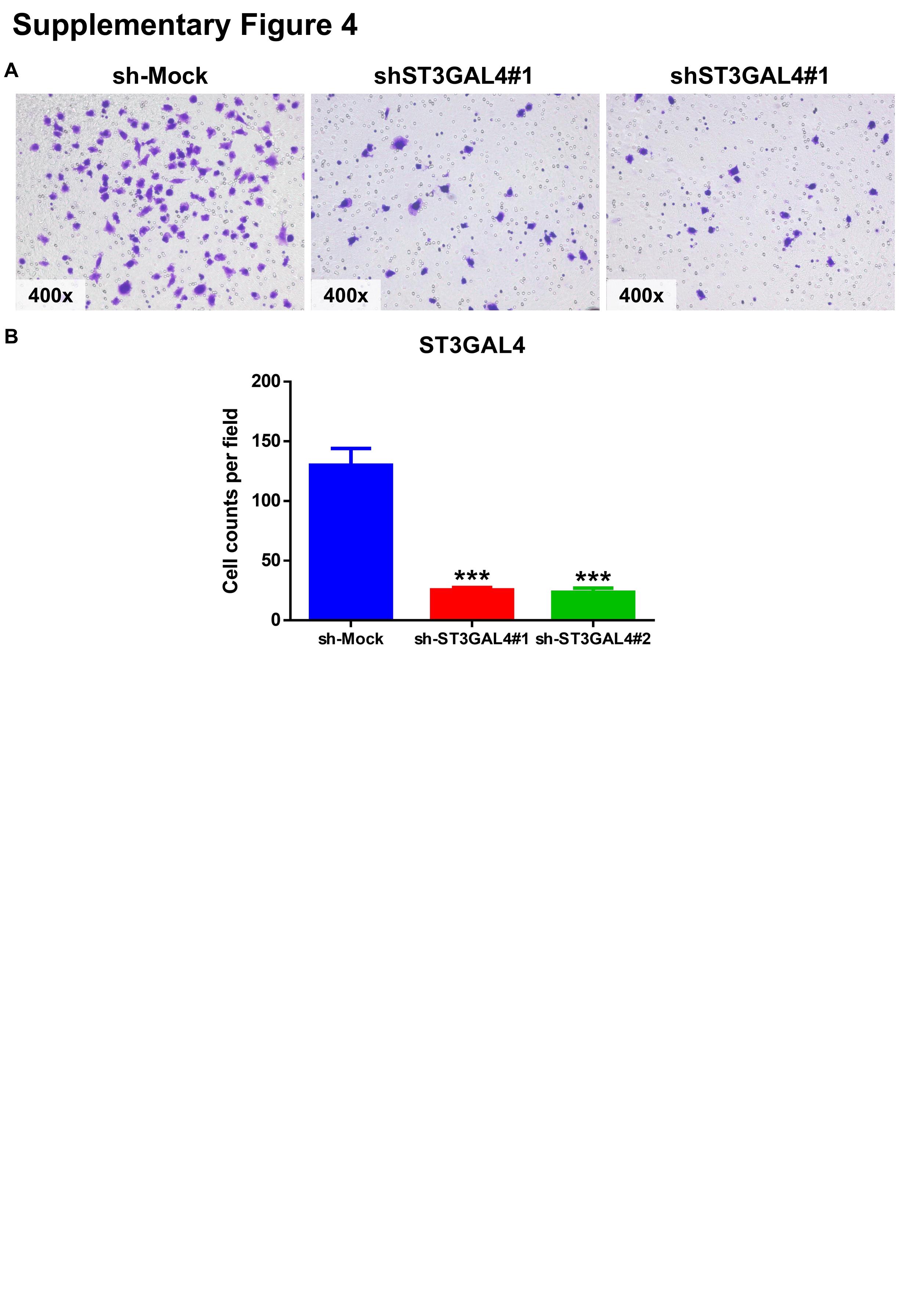

Supplement: Supplementary file 5 — Additional file 5: Figure S4. ST3GAL4 regulates the migration ability of melanoma cells. (A) Knockdown of ST3GAL4 inhibit the migration of SK-MEL-28 cells. Migration assays were performed as described in Methods. Representative images were taken at indicated hours. (B) Bar chart of migration assay. The number of invasive cells per field was calculated, and the data was presented as the means ± SD (n = 4) of each group. The significant difference between cells was evaluated by Student’s t-test. *p < 0.05, **p < 0.01, ***p < 0.001. [file 12935_2021_2164_MOESM5_ESM.jpg]

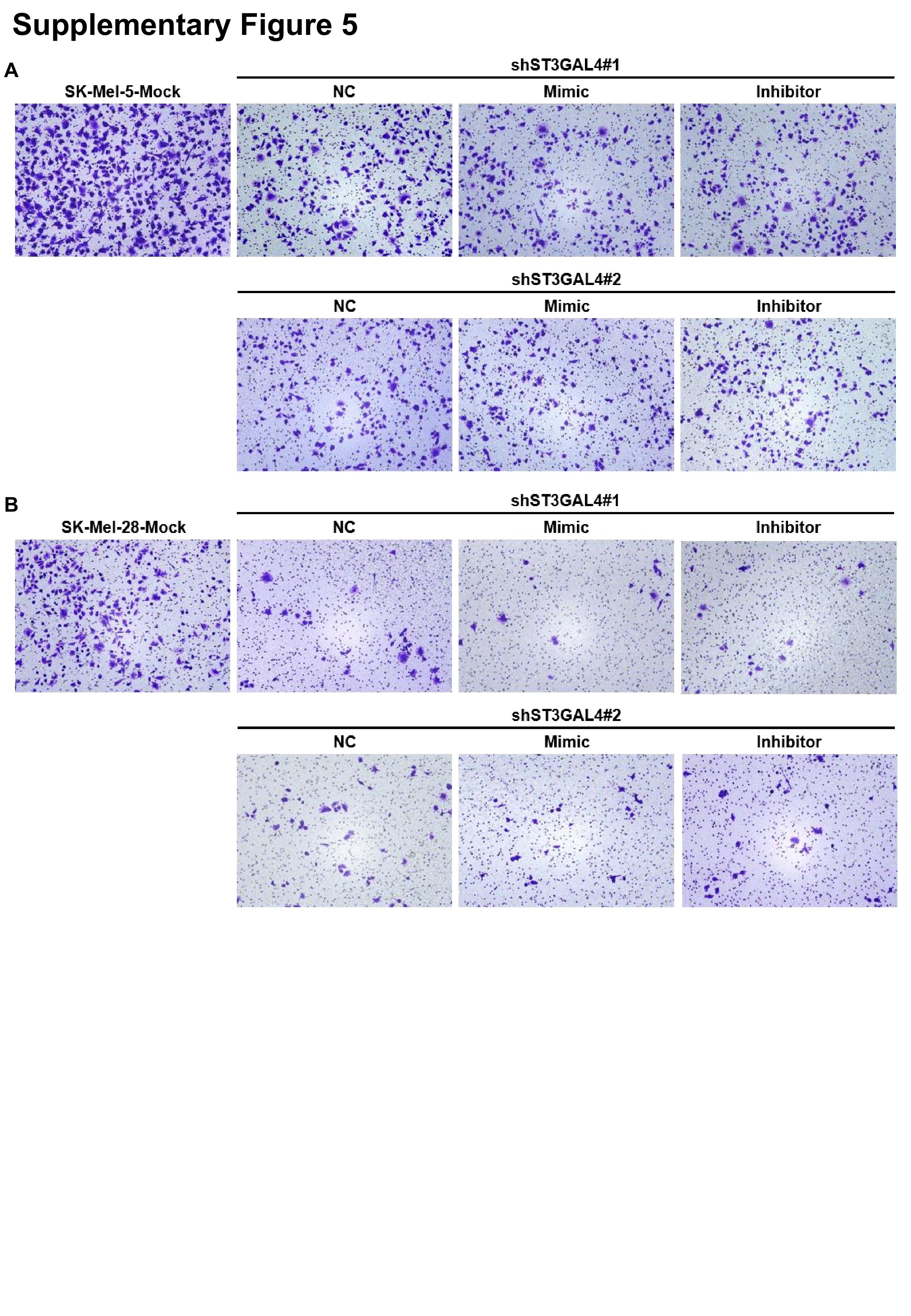

Supplement: Supplementary file 6 — Additional file 6: Figure S5. Knockdown of ST3GAL4 impairs the regulatory role of miR-1180-3p on invasion ability of melanoma cells. (A-B) ST3GAL4-deficient melanoma cells were treated with miR-1180-3p mimics or inhibitors. Representative images of SK-Mel-5 (A) and SK-Mel-28 (B) were taken at indicated hours as described in Methods. [file 12935_2021_2164_MOESM6_ESM.jpg]
